# Usefulness of Pulmonary Artery Pulsatility Index in Predicting Acute Kidney Injury Following Continuous-Flow Left Ventricular Assist Device Implantation in Ambulatory Advanced Heart Failure Patients

**DOI:** 10.3390/jcm15218721

**Published:** 2026-11-08

**Authors:** Cem Doğan, Süleyman Çağan Efe, Gülümser Sevgin Halil, Özgür Yaşar Akbal, Ali Karagöz, Sabit Sarıkaya, Cemalettin Yılmaz, Regayip Zehir, Rezzan Deniz Acar, Mehmet Kaan Kırali

**Affiliations:** 1 Department of Cardiology, Kartal Kosuyolu Training and Research Hospital, Kartal, Istanbul 34865, Türkiye; 2 Department of Cardiovascular Surgery, Kartal Kosuyolu Training and Research Hospital, Kartal, Istanbul 34865, Türkiye; 3 Department of Cardiology, School of Medicine, Yalova University, Yalova 77200, Türkiye

**Keywords:** advanced heart failure, left ventricular assist devices, acute kidney injury, pulmonary artery pulsatility index

## Abstract

**Background:** Acute kidney injury (AKI) is a frequent and serious complication after the implantation of continuous-flow left ventricular assist devices (CF-LVADs), often mediated by right ventricular (RV) dysfunction, systemic venous congestion, and subsequent suboptimal device filling. The pulmonary artery pulsatility index (PAPi) is a hemodynamic parameter reflecting RV function. This study evaluated the predictive value of preoperative PAPi for postoperative AKI in ambulatory advanced heart failure patients. **Methods:** A total of 121 stable, ambulatory advanced heart failure patients (INTERMACS profiles 4–6) who underwent CF-LVAD implantation between 2020 and 2024 were retrospectively analyzed. Preoperative demographic, clinical, and invasive hemodynamic parameters were collected. Postoperative AKI was defined using serum creatinine criteria according to the RIFLE consensus during the first two postoperative days. **Results:** Postoperative AKI developed in 43 patients (35.5%). The mean PAPi value was significantly lower in the AKI group compared with the non-AKI group (1.4 ± 0.4 vs. 3.2 ± 1.2, *p* = 0.001). Preoperative right atrial pressure (RAP) was significantly higher in patients who developed AKI. Receiver operating characteristic (ROC) curve analysis demonstrated modest discrimination, with an area under the curve (AUC) of 0.62 (95% CI: 0.52–0.73). A PAPi cut-off value of 2.1 yielded a sensitivity of 0.82 and a specificity of 0.42. **Conclusions:** In ambulatory advanced heart failure patients undergoing CF-LVAD implantation, a preoperative PAPi value below 2.1 shows an association with a higher risk of postoperative AKI. However, given its modest independent discriminative capacity (AUC 0.62) and potential dependence on venous congestion metrics like RAP, these preliminary findings should be considered exploratory and hypothesis-generating, warranting further external validation in broader cohorts.

## 1. Background

Although survival has increased with recent advancements in the medical management of heart failure, therapeutic options remain limited for patients progressing to advanced stages. Continuous-flow left ventricular assist devices (CF-LVADs) have become a cornerstone of treatment, offering substantial improvements in both survival rates and quality of life for these patient populations [1]. With the evolution of rotary pump technologies, CF-LVAD-related complications have progressively declined, leading to a 5-year average survival rate of approximately 68% and significant symptomatic alleviation to New York Heart Association (NYHA) functional class I or II [2,3,4,5,6].

Despite these technological milestones, patients with advanced heart failure carry an inherent risk of developing renal insufficiency secondary to chronic cardio-renal syndromes, a vulnerability that is acutely exacerbated by major cardiac surgery [7]. Because CF-LVAD implantation is a complex surgical procedure performed in an already compromised cohort, it carries a substantial risk of postoperative acute kidney injury (AKI), which is strongly tied to prolonged hospitalization and poor long-term survival [8]. The reported incidence of AKI after CF-LVAD surgery varies widely in the literature, ranging between 10% and 45% [7,8,9,10,11,12,13,14].

Pathophysiologically, postoperative AKI has been closely linked to right heart failure and elevated central venous pressures [15]. The pulmonary artery pulsatility index (PAPi)—calculated as (systolic pulmonary arterial pressure [sPAP]—diastolic pulmonary arterial pressure [dPAP])/right atrial pressure (RAP)—was initially introduced as a specific predictor of right ventricular (RV) failure in patients experiencing acute inferior myocardial infarction. Subsequently, PAPi has been demonstrated to correlate with clinical outcomes and survival across various spectra of cardiovascular disease, including pulmonary arterial hypertension, asymptomatic dilated cardiomyopathy, advanced heart failure, and the post-heart transplantation setting [8,16].

While the utility of PAPi is well-recognized in acute cardiogenic shock or critically ill populations, its performance specifically within stable, ambulatory cohorts remains less defined. Therefore, this study aimed to investigate the predictive value of the preoperative PAPi value for the development of postoperative AKI exclusively in ambulatory advanced heart failure patients (INTERMACS profiles 4–6) undergoing CF-LVAD implantation.

## 2. Methods

### 2.1. Patient Population and Selection Strategy

This retrospective, observational study evaluated 121 consecutive patients who underwent CF-LVAD implantation at a single tertiary care center between 2020 and 2024. Baseline clinical characteristics, relevant comorbidities, and laboratory parameters were extracted from the institutional electronic health registry system.

The study target was strictly limited to ambulatory advanced heart failure patients. Inclusion criteria were defined as: age ≥18 years, left ventricular ejection fraction (LVEF) ≤ 25%, NYHA functional class II–IV, and an INTERMACS profile of IV–VI. Conversely, patients representing more critical, unstable hemodynamic states were explicitly excluded. Exclusion criteria comprised: age ≥70 years, preoperative inotropic dependency, necessity of an intra-aortic balloon pump (IABP), multi-organ failure, a clinical history of moderate-to-severe intrinsic pulmonary disease, and any standard contraindications to heart transplantation or mechanical circulatory support as specified by the International Society for Heart and Lung Transplantation (ISHLT) guidelines [6].

The restriction to INTERMACS profiles 4–6 was intentionally implemented to evaluate the prognostic value of PAPi within a homogeneous, ambulatory cohort free from the profound, confounding hemodynamic noise of acute cardiogenic shock, pre-existing acute-on-chronic kidney injury, or mechanical circulatory support devices that characterize INTERMACS profiles 1–3.

The study protocol was reviewed and approved by the local Institutional Ethics Board. Informed consent was waived by the committee due to the retrospective design of the study and the exclusive use of fully de-identified patient data.

### 2.2. Right Heart Catheterization and Echocardiography

Invasive hemodynamic assessments were performed via right and left heart catheterization utilizing 6 French pigtail and Swan–Ganz (for pulmonary capillary wedge pressure measurement) catheters under fluoroscopic guidance on the same day as the baseline echocardiographic evaluation. Standardized pressure tracings were recorded for aortic pressure (AoP), systolic pulmonary arterial pressure (PAPs), mean pulmonary arterial pressure (PAPm), diastolic pulmonary arterial pressure (PAPd), right ventricular end-systolic pressure (RVESP), right ventricular end-diastolic pressure (RVEDP), left ventricular end-systolic pressure (LVESP), left ventricular end-diastolic pressure (LVEDP), and right atrial pressure (RAP).

Cardiac output (CO), cardiac index (CI), stroke volume (SV), and stroke volume index (SVI) were calculated using the standard Fick method. Derived hemodynamic indices were calculated according to the following established formulas:

- Trans-pulmonary gradient (TPG) = PAPm − LVEDP.
- Trans-systemic gradient (TSG) = mean AoP − RAP.
- Pulmonary vascular resistance (PVR) = TPG / CO.
- Right ventricular stroke work index (RVSWI) = (PAPm − RAP) × SVI × 0.0136.

Using the baseline right-heart pressures, the primary hemodynamic predictor, PAPi, was calculated as:

PAPi = PAPs − PAPd/RAP

Transthoracic echocardiography was conducted concurrently to document left ventricular end-diastolic and end-systolic diameters, LVEF, TAPSE (tricuspid annular plane systolic excursion), and left atrial diameter.

### 2.3. Outcome Definition

Serum creatinine levels were systematically recorded at baseline prior to CF-LVAD implantation and for two consecutive days immediately following surgery. Postoperative AKI was defined using the serum creatinine criteria of the RIFLE consensus framework [17]. The primary outcome was defined as the occurrence of RIFLE Class R or greater, characterized by a >50% increase in serum creatinine levels from the baseline value within the first 48 postoperative hours.

### 2.4. Statistical Analysis

Continuous variables are presented as mean ± standard deviation (SD) or median with interquartile range (IQR) based on normality testing, while categorical data are expressed as absolute numbers and percentages. The study population was stratified into two distinct cohorts based on the development of postoperative AKI (the AKI group versus the non-AKI group).

Candidate predictor variables are described below:

- Base model: Age, preoperative creatinine level, and PAPi were included in the model as continuous variables.
- Model-1: base model + left ventricular ejection fraction: albumin level.
- Model-2: model-1 + N terminal B-type natriuretic peptide: diabetes mellitus.

The primary candidate predictor variable of interest was the preoperative PAPi. Due to a low event-per-variable rate (approximately 5 events per variable), standard multivariable logistic regression can yield biased estimates. To mitigate this and prevent overfitting, Firth’s penalized logistic regression was performed using the logistf package in R. This approach applies a penalty to the likelihood function to ensure reliable parameter estimation in small samples with low event rates.

Model calibration and internal validation were rigorously assessed using the rms package via a bootstrapping procedure with 1000 resamples to compute optimism-corrected performance metrics. The model exhibited an optimism-corrected Somers’ D_xy_ change of 0.05 and an R^2^ shift of 0.039.

Missing data were thoroughly evaluated before modeling. If data were determined to be Missing at Random (MAR), missing numeric predictors (affecting <10% of observations) and missing binary predictors (affecting <5% of observations) were imputed using the aregImpute function from the Hmisc package in R, which leverages additive regression, bootstrapping, and predictive mean matching. Observations with data missing not at random (MNAR) or those missing the primary outcome variable (postoperative AKI) were excluded from the final analysis.

Receiver operating characteristic (ROC) curve analysis was executed to evaluate the discriminative power of PAPi and to identify the optimal cut-off value using the Youden index. Area under the curve (AUC) metrics are reported alongside their respective 95% confidence intervals (CI). Comparisons between multiple ROC curves (e.g., comparing PAPi against RAP alone) were performed using DeLong tests, with *p*-values adjusted for multiple testing using the Holm method. All statistical analyses were conducted using R software (the 4.2 version), and a two-sided *p*-value < 0.05 was considered statistically significant.

## 3. Result

In this study, a total of 121 advanced heart failure patients who had an implanted CF-LVAD were included. Postoperative AKI was seen in 43 (35.5%) patients, none of whom needed permanent dialysis. Baseline characteristics of patients without (N0N-AKI group) and with (AKI group) AKI are given in Table 1. Mean age was not statistically different between groups (48.7 ± 9.4 versus 52.1 ± 8.4, *p* = 0.051). The study population was male-dominant (113, 93.4%), and gender was not statistically different between groups [72 (92.3%) versus 41 (95.3%), *p* = 0.519].

There was no difference between the groups in terms of the presence of coronary artery disease [40.0 (51.3%) versus 29.0 (67.4%) *p* = 0.086] and comorbidities except diabetes mellitus, 11.0 (14.1%) versus 15.0 (34.9%) *p* = 0.008 [hypertension 11.0 (14.1%) versus 12.0 (27.9%), smoking 31 (39.7%) versus 22 (51.2%), chronic obstructive pulmonary disease 6.0 (7.7%) versus 1.0 (2.3%), and atrial fibrillation 15.0 (19.2%) versus 8.0 (18.6%) all >0.05]. In laboratory tests, the preoperative creatinine level (1.0 ± 0.4 versus 1.1 ± 0.4 mg/dL *p* = 0.130) and estimated Glomerular Filtration Rate (calculated using the CKD-EPI equation) (eGFR) (88.4 ± 24.2 vs. 96.5 ± 23.7 mL/min/1.73 m^2^, *p* = 0.106), were similar in both groups, but the NT-proBNP level of the AKI group was higher than the non-AKI group (2517.0 ± 3087.4 versus 1587.9 ± 1888.8 *p* = 0.042). The echocardiographic characteristics of the patients were similar in the two groups, and they are summarized in Table 1.

The invasive hemodynamic characteristics of the patients are summarized in Table 2. The cardiac index of the non-AKI group was higher than that of the AKI group (1.7 ± 0.3 versus 1.6 ± 0.3, *p* = 0.006). PAPi, an index of right ventricular function, was lower in the AKI group than in the non-AKI group (1.4 ± 0.4 versus 3.2 ± 1.2, *p* = 0.001), and RAP was also higher in the AKI group (14.6 ± 6.1 versus 8.2 ± 3.5, *p* = 0.001).

A base model was used to seek the relationship between candidate predictors and the presence of AKI. Among the variables, a decrease in PAPi [OR: 0.51 (0.28–0.89)] was independently associated with the presence of AKI, with the other variables shown in Table 3. Also, we assessed the robustness of PAPi with model-1 and model-2. In model-1 and model-2, although other parameters such as NT-proBNP, LVEF, and hemoglobin were added to PAPi, it was still found to be significant, which shows the robustness of PAPi.

**Figure 1:**
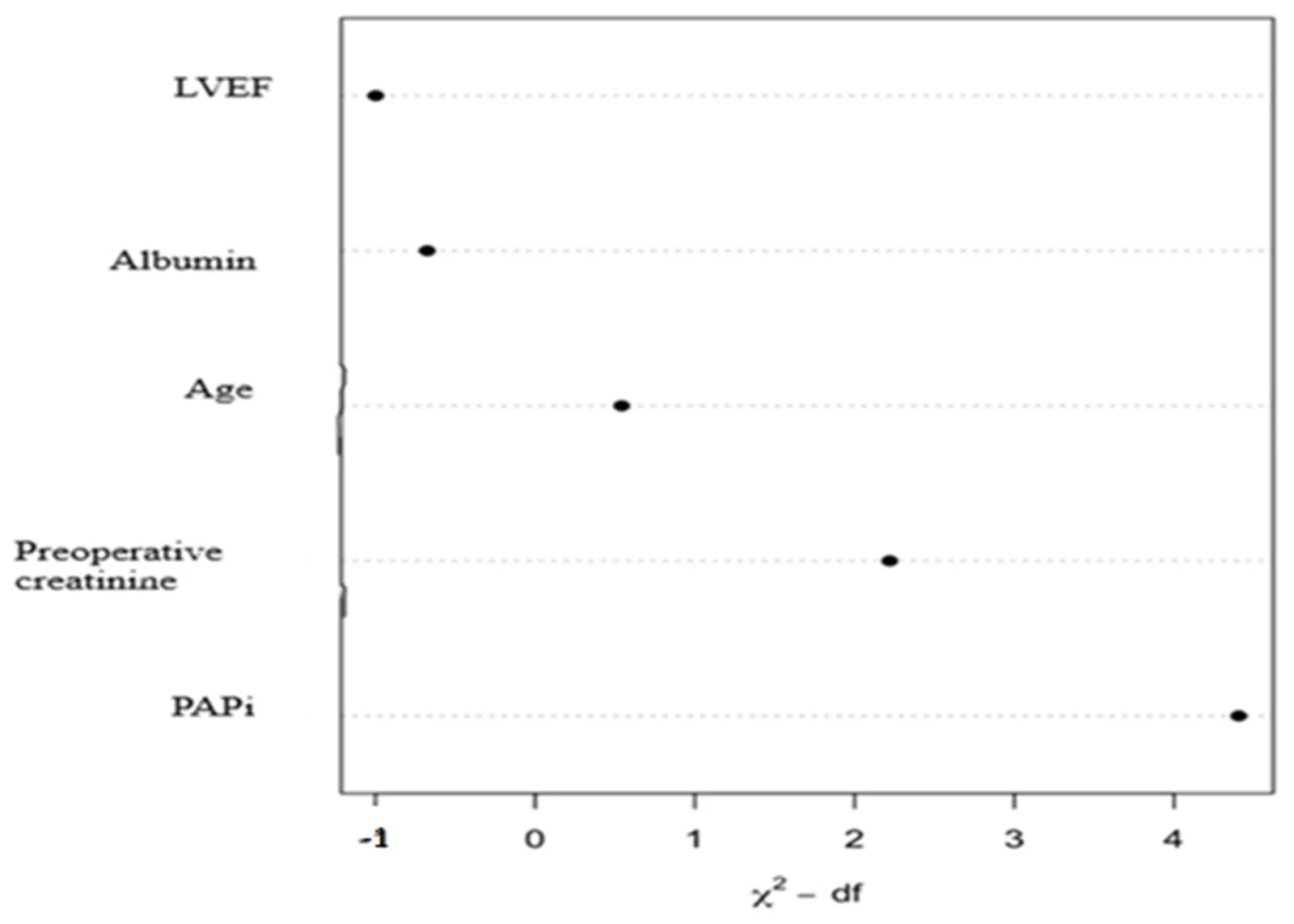
Importance of individual predictors. The relative importance of each predictor in our model was calculated as the partial χ^2^ statistic for that predictor divided by the model’s total χ^2^ statistic.

In Figure 1, we showed the relative importance of each predictor in model-2. PAPi was ranked as the predictor that contributed the most to the presence of AKI. Also, the second most important variable was the preoperative creatinine level.

**Figure 2:**
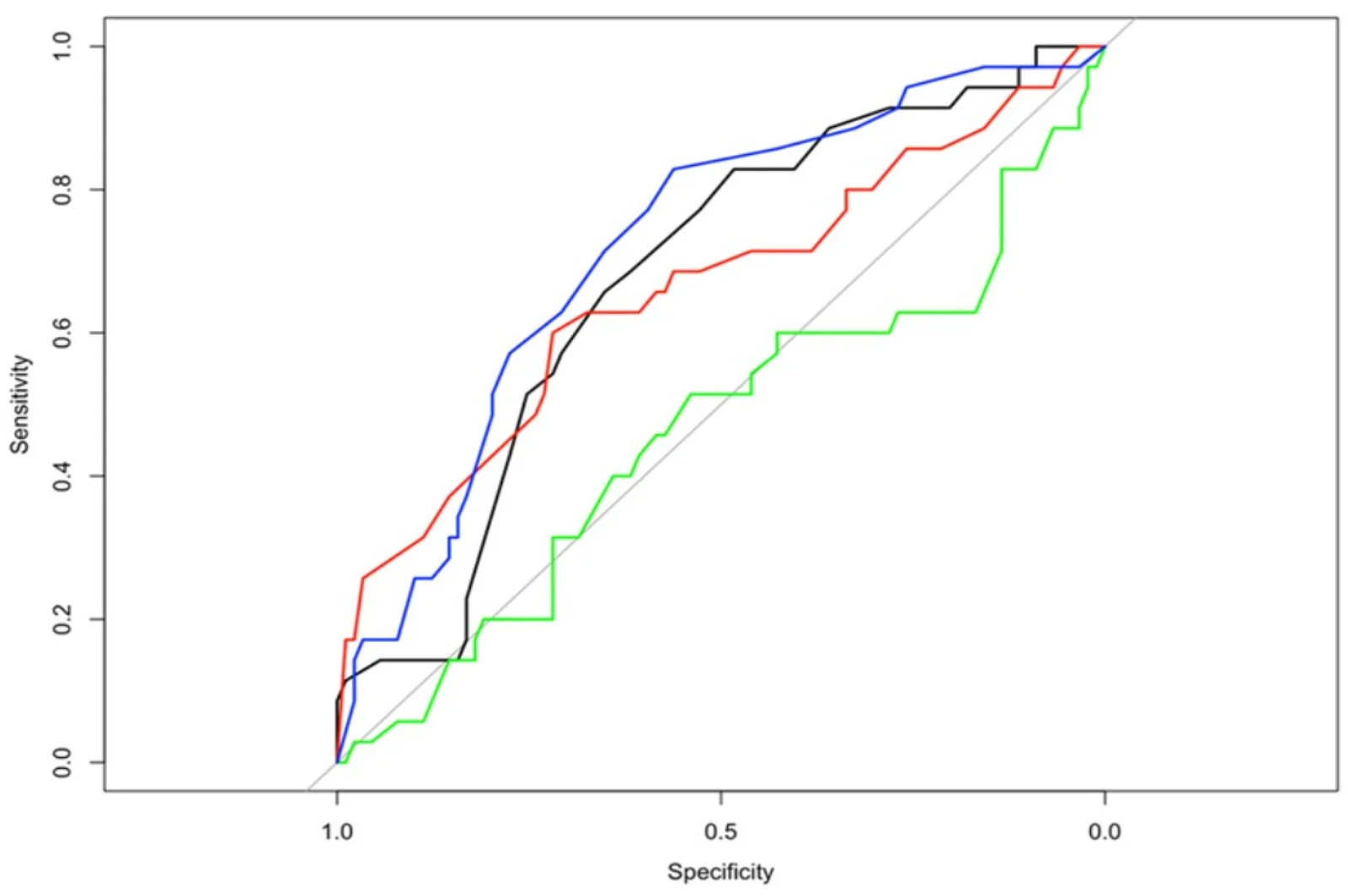
ROC analysis showed that the cut-off value of 2.1 for PAPi had a sensitivity of 0.82 and 0.42 specificity, with the largest AUC value of 0.62, which was larger than that of the other variables (PAPi, pulmonary artery pulsatility index (blue line); RAP; right atrial pressure (black line); SBP, systolic blood pressure (green line); DBP, diastolic blood pressure (red line)).

**Figure 3:**
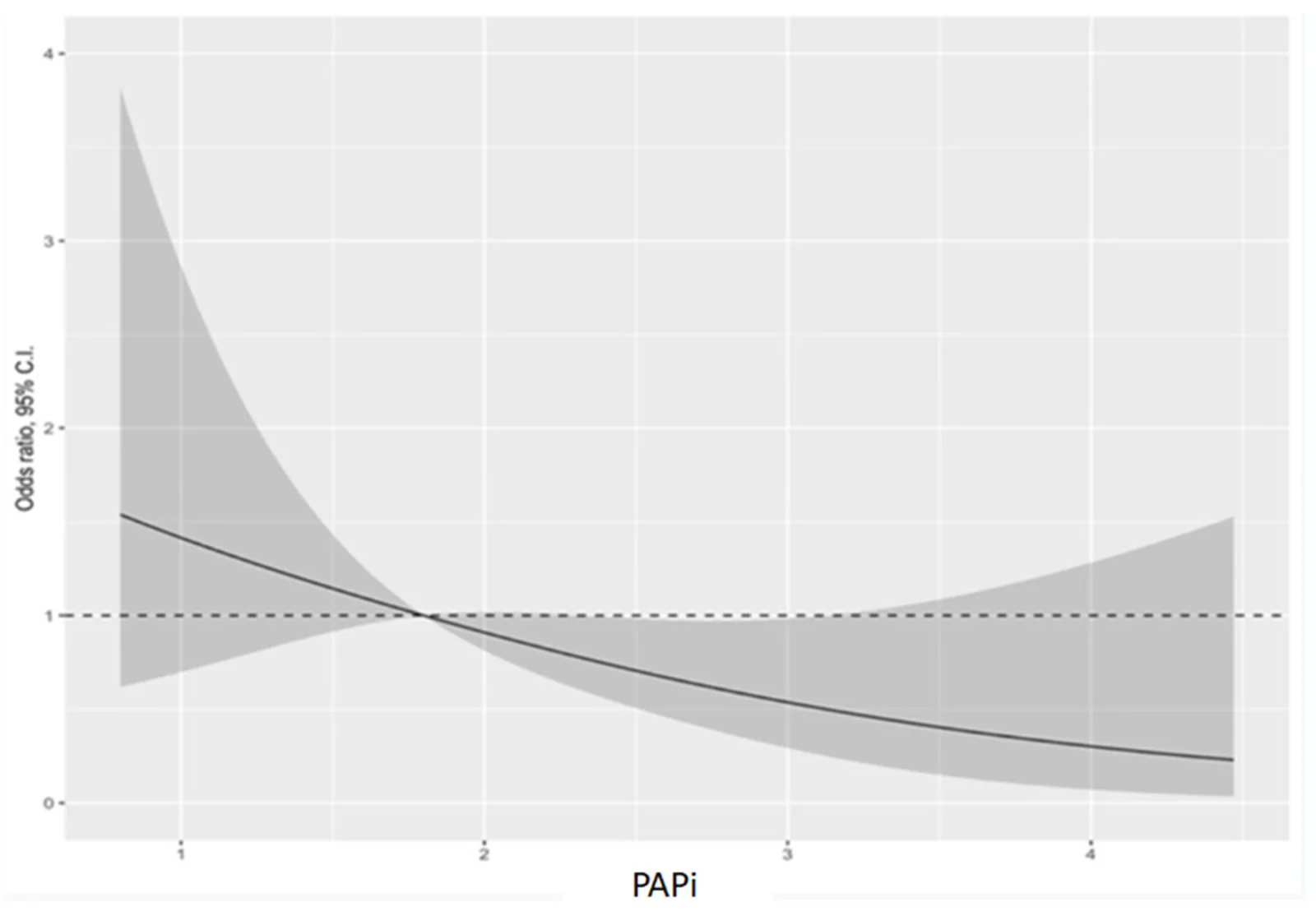
Relation of odds of acute kidney injury (AKI) with pulmonary artery pulsatility index (PAPi).

The ROC-AUC of the four parameters [PAPi, RAP, systolic blood pressure (SBP), and diastolic blood pressure (DBP)] were compared by the DeLong method. The ROC-AUC of the model including PAPi was significantly higher than that of models including RAP, SBP, and DBP (*p*-values 0.04, 0.005, and 0.001, respectively). The PAPi cut-off values for the development of AKI were evaluated by the Youden method, and the best cut-off value was identified as 2.1 (sensitivity of 0.82 and specificity of 0.42; AUC 0.62, 0.52–0.73 95% CI). (Figure 2). In Figure 3, the probability of AKI was plotted according to PAPi, which showed that after decreasing to 1.8, the probability of AKI increases linearly.

## 4. Discussion

There are two important results of this study: 1—PAPi is an independent predictor for the development of AKI after CF-LVAD implantation; 2—The PAPi < 2.1 cut-off value is associated with postoperative AKI.

With the introduction of magnetic levitated centrifugal pumps and their proven successful results, CF-LVAD has become an important treatment option for end-stage heart failure patients with heart transplantation [18]. Some of those in this group of patients who are LVAD candidates had renal dysfunction secondary to heart failure in the preoperative period [7]. As with all cardiovascular surgeries, there is a risk of developing postoperative AKI in CF-LVAD implantation, and this is associated with mortality [19,20]. Depending on the criteria used, the rate of AKI in this patient group varies between 10% and 45% [7,8,9,10,11,12,13,14]. Postoperative AKI was seen in 35.5% of our patients, and none of them needed permanent dialysis. This finding should be interpreted with caution, as individuals with severe, pre-existing chronic renal failure are generally not considered suitable candidates for durable CF-LVAD therapy. The fact that our patients who developed AKI had higher NT-pro-BNP levels and lower CIs suggests that impaired renal perfusion in the perioperative period increases the risk, and this shows the importance of planning CF-LVAD implantation before the development of extreme hemodynamic instability.

**Figure 4:**
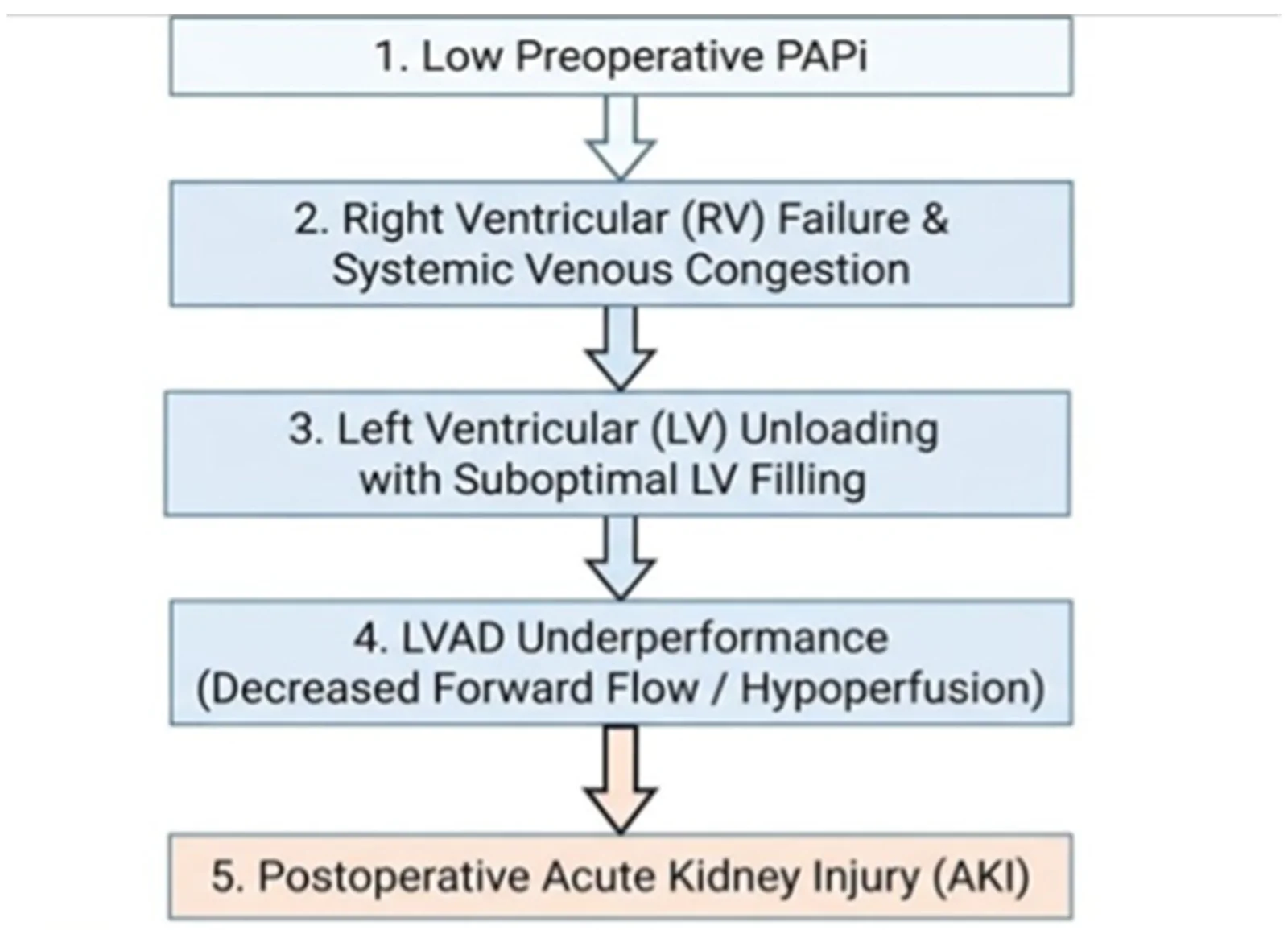
Flow chart showing the relationship between PAPi and the development of AKI.

When we consider the pathophysiological mechanisms, the relationship between preoperative PAPi and postoperative AKI is mediated through right ventricular (RV) congestion and failure, which directly impacts left ventricular (LV) filling and subsequent continuous-flow LVAD performance (Figure 4). After LVAD implantation, increased RV preload and decreased contractility to cope with the increased cardiac output cause RV dysfunction [21]. Previous studies have shown that when RV dysfunction develops, RV filling pressure increases disproportionately to LV filling pressure, causing hypotension and an increase in central venous pressure, leading to the development of AKI [16].

PAPi has already been evaluated preoperatively in patients requiring LVAD. Several studies have shown the correlation between a decreased PAPi and the occurrence of postoperative RV dysfunction [22,23,24]. In our study, we used PAPi as a marker of RV dysfunction to determine postoperative AKI. PAPi maintains its significant relationship with AKI even when other parameters are added in both the base model and models 1 and 2. The AKI group demonstrated significantly lower cardiac output/index alongside elevated RAP. Although systolic PA pressures were similar, the narrower PA pulse pressure (sPAP − dPAP) in the AKI group reflects a state of decreased forward pulmonary blood flow combined with systemic venous congestion (elevated RAP). Therefore, PAPi acts as a comprehensive index capturing both components (low forward flow and high backward congestion), making it a superior surrogate for right ventricular–pulmonary arterial (RV-PA) coupling rather than a simple pressure metric. There is no other study showing the relationship of PAPi with postoperative AKI in the patient group undergoing CF-LVAD implantation. In previous studies, many parameters and scores have been developed to evaluate RV functions in this patient group. PAPi was first reported to be a powerful predictor of RV failure in patients with acute inferior myocardial infarction by Korabathina et al. [25]. Also, studies have shown that PAPi is associated with mortality in patients with pulmonary arterial hypertension, dilated cardiomyopathy, advanced HF, and heart transplantation [26,27,28].

When we performed the ROC curve analysis of RAP, SBP, and DBP together with PAPi, which we think may be effective in predicting the development of postoperative AKI, we saw that the best AUC value was for PAPi. From a clinical utility perspective, it is worth noting that while RAP can be easily estimated non-invasively via echocardiography or monitored via a central venous line, it only provides a static assessment of congestion. In our study, PAPi demonstrated superior discriminative capacity over RAP alone (*p* = 0.04 via DeLong test). Given that right heart catheterization is an indispensable and mandatory component of the standard preoperative evaluation for durable CF-LVAD candidates to rule out irreversible pulmonary hypertension, the calculation of PAPi utilizes already available data without exposing the patient to additional invasive risks. In predicting the development of postoperative AKI, a cut-off value of PAPi < 2.1 had a sensitivity of 0.82 and a specificity of 0.42 (AUC: 0.62, 0.52–0.73 95% CI) (Figure 2). Also, the probability of AKI was plotted according to PAPi, which showed that Also, the probability of AKI was plotted according to PAPi, which showed that after decreas to a 1.8 probabilty of AKI increased linearly (Figure 3). Physiologically, since RAP constitutes the denominator of the PAPi equation, a low PAPi score may largely reflect elevated RAP (systemic venous congestion) rather than isolated RV systolic contractile failure. This is supported by our finding that TAPSE—a direct metric of RV longitudinal systolic function—was only marginally and non-significantly higher in the AKI group (1.5 vs. 1.4 cm, *p* = 0.055). This underscores that PAPi is a preload-dependent index. Nonetheless, our comparative analysis demonstrated that PAPi offers incremental predictive value for post-LVAD AKI beyond RAP alone, suggesting that the combination of pulsatile RV stroke work and venous congestion captured by PAPi is a superior predictor than pressure metrics alone.

In the studies, PAPi cut-off values varied widely, and different thresholds were accepted for different populations (PAPi ≤ 0.9 in patients with cardiogenic shock, PAPi < 1.85 in patients undergoing LVAD, and PAPi < 3.65 in patients with advanced HF) [22,25,29]. The cut-off values we obtained are consistent with the values in these studies examining similar patient groups and indicate that they can be used to predict postoperative AKI after CF-LVAD implantation.

## 5. Limitations

A significant limitation of this study is the restriction of the study population to INTERMACS profiles 4–6, thereby excluding the critically ill patients (INTERMACS 1–3) who were inotrope- or IABP-dependent. Preoperative hemodynamic assessment and the pulmonary artery pulsatility index (PAPi) are arguably of greatest clinical significance in this high-risk cohort. However, we excluded INTERMACS 1–3 patients because aggressive inotropic support, active mechanical circulatory assistance, and acute cardiogenic shock heavily confound baseline PAPi measurements and rapidly alter renal perfusion. By focusing on a more stable, elective cohort (INTERMACS 4–6), we aimed to evaluate the independent predictive value of PAPi for post-LVAD acute kidney injury. Consequently, our findings cannot be generalized to sicker, hemodynamically unstable patients, and future studies are required to validate the role of PAPi in the INTERMACS 1–3 population. Furthermore, our study is limited by the lack of granular data on major perioperative confounders, including cardiopulmonary bypass time, perioperative blood transfusions, and concomitant valve surgeries. These unmeasured confounders heavily influence both RV performance and renal perfusion, and their absence should be considered when interpreting our regression models. In our institution, baseline diagnostic right-heart catheterization (RHC) in stable, ambulatory advanced heart failure patients (INTERMACS 4–6) is routinely performed using standard catheters (such as 6F pigtail or multipurpose catheters) via femoral or jugular access rather than maintaining a continuous Swan–Ganz catheter in the intensive care setting; we used the Swan–Ganz catheter only for measurement of PCWP. Cardiac output was derived via the standardized Fick method using measured oxygen consumption or established nomograms. We acknowledge that severe mitral or tricuspid regurgitation can introduce errors in Fick-derived calculations. Acute kidney injury (AKI) was defined using the historical RIFLE criteria based on serum creatinine changes within the first 48 h postoperatively, as per our institutional registry design. We acknowledge that the lack of urine-output criteria and the short evaluation window represent a methodological limitation. Consequently, cases of milder, non-oliguric, or later-onset AKI may have been under-captured, which could potentially affect the reported baseline incidence of AKI in our cohort.

## 6. Conclusions

AKI developing after CF-LVAD implantation is closely related to RV dysfunction and is an important cause of morbidity and mortality. A low preoperative pulmonary artery pulsatility index (PAPi) is a valuable and independent predictor of acute kidney injury following continuous-flow LVAD implantation, specifically in ambulatory advanced heart failure patients (INTERMACS profiles 4–6). Extrapolating these findings to critically ill, inotrope- or IABP-dependent patients (INTERMACS 1–3) requires extreme caution, and further validation in this high-risk population is warranted.

## Figures and Tables

**Table 1:** Demographic, clinical, and echocardiographic characteristics of AKI and non-AKI patients.

Variable	NON-AKI (78)	AKI (43)	*p*
Age, years	48.7 ± 9.4	52.1 ± 8.4	0.051
Diabetes Mellitus, *n* (%)	11.0 (14.1%)	15.0 (34.9%)	0.448
Hypertension, *n* (%)	11.0 (14.1%)	12.0 (27.9%)	0.064
Gender (Male), *n* (%)	72.0 (92.3%)	41.0 (95.3%)	0.519
Smoking, *n* (%)	31.0 (39.7%)	53.0 (43.8%)	0.226
COPD, *n* (%)	6.0 (7.7%)	1.0 (2.3%)	0.226
CAD, *n* (%)	40.0 (51.3%)	29.0 (67.4%)	0.086
Atrial Fibrillation, *n* (%)	15.0 (19.2%)	8.0 (18.6%)	0.933
Preoperative creatinine, mg/dL	1.0 ± 0.4	1.1 ± 0.4	0.130
Sodium, mEq/L	135.0 ± 4.1	136.4 ± 3.9	0.067
Potasium, mEq/L	4.4 ± 0.8	4.4 ± 0.6	0.753
Uric Acid, mg/dL	8.7 ± 2.1	8.4 ± 2.6	0.441
AST, mEq/L	35.2 ± 28.7	29.1 ± 16.5	0.204
ALT, mEq/L	41.0 ± 59.8	31.1 ± 31.5	0.314
NT-proBNP, pg/mL	1587.9 ± 1888.8	2517.0 ± 3087.4	0.042
Albumin, mg/dL	3.9 ± 0.5	3.9 ± 0.5	0.695
Total Cholesterol, mg/dL	147.8 ± 48.6	178.2 ± 56.8	0.002
WBC, μL	8.8 ± 2.9	8.3 ± 2.3	0.346
Hemoglobin, g/dL	12.8 ± 1.8	12.6 ± 2.0	0.617
Platelet, μL	254.1 ± 82.4	236.2 ± 76.3	0.204
Height, cm	171.7 ± 9.1	170.9 ± 6.8	0.600
Weight, kg	77.2 ± 14.8	77.2 ± 13.7	0.981
BMI, kg/m^2^	26.5 ± 4.8	25.5 ± 4.7	0.245
LVEF, %	19.7 ± 4.5	19.7 ± 2.9	0.905
LVEDD, cm	7.2 ± 0.9	7.1 ± 0.7	0.472
LVESD, cm	7.1 ± 5.8	6.3 ± 3.8	0.351
PAPs, mmHg	50.7 ± 12.9	50.9 ± 15.2	0.093
TAPSE, cm	1.4 ± 0.3	1.5 ± 0.4	0.055
LA, cm	5.1 ± 0.7	4.9 ± 0.7	0.352

Values were presented as mean ± SD, % of cohort. AKI, acute kidney injury; ALT, alanine aminotransferase; AST, aspartate aminotransferase; BMI, body mass index; CAD, coronary artery disease; COPD, chronic obstructive pulmonary disease; LA, left atrial dimension; LVEDD, left ventricle end-diastolic dimension; LVEF, left ventricular ejection fraction; LVESD, left ventricle end-systolic dimension; NT-BNP, N Terminal brain natriuretic peptide; PAPs, systolic pulmonary artery pressure; TAPSE, tricuspid annular plane systolic excursion; WBC, white blood cell.

**Table 2:** Cardiac catheterization values of AKI and non-AKI patients.

Variable	Non-AKI (78)	AKI (43)	*p*
CO, L/min	3.3 ± 0.8	3.0 ± 0.6	0.036
CI, L/min/m^2^	1.7 ± 0.3	1.6 ± 0.3	0.006
SBP, mmHg	106.8 ± 18.7	101.3 ± 19.2	0.131
DBP, mmHg	64.7 ± 13.9	66.2 ± 13.8	0.574
PCWP, mmHg	24.4 ± 8.1	25.3 ± 7.7	0.541
PAPs, mmHg	60.5 ± 14.9	56.4 ± 16.3	0.181
meanPAP, mmHg	37.3 ± 9.8	37.5 ± 10.6	0.928
PAPi	3.2 ± 1.2	1.4 ± 0.4	0.001
RAP	8.2 ± 3.5	14.6 ± 6.1	0.001

Values were presented as mean ± SD, % of cohort. AKI, acute kidney injury; CI, cardiac index; CO, cardiac output; DBP, diastolic blood pressure; PAP, pulmonary artery pressure; PAPi, pulmonary artery pulsatility index; PCWP, pulmonary capillary wedge pressure; RAP, right atrial pressure; SBP, systolic blood pressure.

**Table 3:** The performance of the base model and reduced model is demonstrated in the Table.

Variable	Base Model		Model-1		Model-2	
	OR CI:95%	*p* Value	OR CI:95%	*p* Value	OR CI:95%	*p* Value
Age (from 44 to 56)	1.51 (0.81–2.81)	0.18	1.48 (0.79–2.76)	0.77	1.52 (0.76–3.06)	0.23
Preop. Creat. (0.78 to 1.13)	0.61 (0.37–1.02)	0.06	0.62 (0.37–1.04)	0.21	0.57 (0.32–0.99)	0.04
PAPi (1.2 to 2.3)	0.50 (0.28–0.89)	0.01	0.51 (0.29–0.90)	0.02	0.50 (0.28–0.89)	0.02
Albumin (3.6 to 4.2)			0.86 (0.52–1.42)	0.56	0.85 (0.48–1.51)	0.57
LVEF (15 to 20%)			0.51 (0.29–0.90)	0.02	1.07 (0.59–1.93)	0.82
NT-proBNP (477 to 2633)					0.04 (0.19–0.81)	0.01
Hemoglobin (11.4 to 13.7)					0.82 (0.42–1.59)	0.54
Diabetes mellitus (Yes/No)					2.86 (0.92–8.91)	0.06

Likelihood ratio and adjusted R^2^ values for the reduced model were slightly lower than those of the full model. Discriminative indices, such as the Harrel C-index, of the reduced model were also slightly lower than those of the full model. (C-statistic measures the discriminative ability of the model, and values closer to 1.0 are better), with the others shown in the Table. LVEF, left ventricular ejection fraction; NT-BNP, N Terminal brain natriuretic peptide; PAPi, pulmonary artery pulsatility index.

## Data Availability

The data that support the findings of this study are available from the corresponding author upon reasonable request. The data are not publicly available due to patient privacy and ethical restrictions.

## References

[1] Mehra M.R. (2019). The burden of haemocompatibility with left ventricular assist systems: A complex weave. Eur. Heart J..

[2] Slaughter M.S., Rogers J.G., Milano C.A., Russell S.D., Conte J.V., Feldman D., Sun B., Tatooles A.J., Delgado R.M.I., Long J.W. (2009). Advanced heart failure treated with continuous-flow left ventricular assist device. N. Engl. J..

[3] Muslem R., Caliskan K., Leebeek F.W.G. (2018). Acquired coagulopathy in patients with left ventricular assist devices. J. Thromb. Haemost..

[4] Uriel N., Pak S.-W., Jorde U.P., Jude B., Susen S., Vincentelli A., Ennezat P.-V., Cappleman S., Naka Y., Mancini D. (2010). Acquired von Willebrand syndrome after continuous-flow mechanical device support contributes to a high prevalence of bleeding during long-term support and at the time of transplantation. J. Am. Coll. Cardiol..

[5] Heilmann C., Geisen U., Beyersdorf F., Nakamura L., Trummer G., Berchtold-Herz M., Schlensak C., Zieger B. (2011). Acquired Von Willebrand syndrome is an early-onset problem in ventricular assist device patients. Eur. J. Cardiothorac. Surg..

[6] Pagani F.D., Aaronson K.D., Kormos R., Mann D.L., Spino C., Jeffries N., Taddei-Peters W.C., Mancini D.M., McNamara D.M., Grady K.L. (2016). The NHLBI REVIVE-IT study: Understanding its discontinuation in the context of current left ventricular assist device therapy. J. Heart Lung Transplant..

[7] Brisco M.A., Kimmel S.E., Coca S.G., Putt M.E., Jessup M., Tang W.W., Parikh C.R., Testani J.M. (2014). Prevalence and prognostic importance of changes in renal function after mechanical circulatory support. Circ. Heart Fail..

[8] Sandner S.E., Zimpfer D., Zrunek P., Rajek A., Schima H., Dunkler D., Grimm M., Wolner E., Wieselthaler G.M. (2009). Renal function and outcome after continuous flow left ventricular assist device implantation. Ann. Thorac. Surg..

[9] Hasin T., Topilsky Y., Schirger J.A., Li Z., Zhao Y., Boilson B.A., Clavell A.L., Rodeheffer R.J., Frantz R.P., Edwards B.S. (2012). Changes in renal function after implantation of continuous-flow left ventricular assist devices. J. Am. Coll. Cardiol..

[10] Park S.J., Milano C.A., Tatooles A.J., Rogers J.G., Adamson R.M., Steidley D.E., Ewald G.A., Sundareswaran K.S., Farrar D.J., Slaughter M.S. (2012). Outcomes in advanced heart failure patients with left ventricular assist devices for destination therapy. Circ. Heart Fail..

[11] Patel A.M., Adeseun G.A., Ahmed I., Mitter N., Rame J.E., Rudnick M.R. (2013). Renal failure in patients with left ventricular assist devices. Clin. J. Am. Soc. Nephrol..

[12] Yoshioka D., Sakaguchi T., Saito S., Miyagawa S., Nishi H., Yoshikawa Y., Fukushima S., Saito T., Daimon T., Ueno T. (2012). Predictor of early mortality for severe heart failure patients with left ventricular assist device implantation: Significance of INTERMACS level and renal function. Circ. J..

[13] Starling R.C., Naka Y., Boyle A.J., Gonzalez-Stawinski G., John R., Jorde U., Russell S.D., Conte J.V., Aaronson K.D., McGee E.C. (2011). Results of the post-U.S. Food and Drug Administration-approval study with a continuous flow left ventricular assist device as a bridge to heart transplantation: A prospective study using the INTERMACS (Interagency Registry for Mechanically Assisted Circulatory Support). J. Am. Coll. Cardiol..

[14] Alba A.C., Rao V., Ivanov J., Ross H.J., Delgado D.H. (2009). Predictors of acute renal dysfunction after ventricular assist device placement. J. Card. Fail..

[15] Ross D.W., Stevens G.R., Wanchoo R., Majure D.T., Jauhar S., Fernandez H.A., Merzkani M., Jhaveri K.D. (2018). Left Ventricular Assist Devices and the Kidney. Clin. J. Am. Soc. Nephrol..

[16] Schramm R., Kirchner J., Ibrahim M., Rojas S.V., Morshuis M., Rudolph V., Gummert J.F., Fox H. (2024). Pulmonary Vascular Resistance to Predict Right Heart Failure in Patients Undergoing Left Ventricular Assist Device Implantation. J. Clin. Med..

[17] Bellomo R., Ronco C., Kellum J.A., Mehta R.L., Palevsky P. (2004). Acute Dialysis Quality Initiative workgroup. Acute renalfailure—Definition, outcome measures, animal models, fluid therapy and information technology needs: The Second International Consensus Conference of the Acute Dialysis Quality Initiative (ADQI) Group. Crit. Care.

[18] Javorski M.J., Zaki A., Abas M., Elgharably H., Attia T.S. (2021). The current era of left ventricular assist devices. Future Cardiol..

[19] Naik A., Akhter S.A., Fedson S., Jeevanandam V., Rich J.D., Koyner J.L. (2014). Acute kidney injury and mortality following ventricular assist device implantation. Am. J. Nephrol..

[20] Borgi J., Tsiouris A., Hodari A., Cogan C.M., Paone G., Morgan J.A. (2013). Significance of postoperative acute renal failure after continuous-flow left ventricular assist device implantation. Ann. Thorac. Surg..

[21] Kormos R.L., Teuteberg J.J., Pagani F.D., Russell S.D., John R., Miller L.W., Massey T., Milano C.A., Moazami N., Sundareswaran K.S. (2010). Right ventricular failure in patients with the HeartMate II continuous-flow left ventricular assist device: Incidence, risk factors, and effect on outcomes. J. Thorac. Cardiovasc. Surg..

[22] Morine K.J., Kiernan M.S., Pham D.T., Paruchuri V., Denofrio D., Kapur N.K. (2016). Pulmonary Artery Pulsatility Index Is Associated With Right Ventricular Failure After Left Ventricular Assist Device Surgery. J. Card. Fail..

[23] Essandoh M., Kumar N., Hussain N., Dalia A.A., Wang D., Al-Qudsi O., Wilsak D., Stahl D., Bhatt A., Awad H. (2022). Pulmonary artery pulsatility index as a predictor of right ventricular failure in left ventricular assist device recipients: A systematic review. J. Heart Lung Transplant..

[24] Kumar S., Rahman A., Josephs J.S., Saeed O., Patel S., Murthy S., Shin J.J., Forest S., Vukelic S., Goldstein D.J. (2019). Trend in pulmonary artery pulsatility index pre- to post-LVAD implantation. J. Heart Lung Transplant..

[25] Korabathina R., Heffernan K.S., Paruchuri V., Patel A.R., Mudd J.O., Prutkin J.M., Orr N.M., Weintraub A., Kimmelstiel C.D., Kapur N.K. (2012). The pulmonary artery pulsatility index identifies severe right ventricular dysfunction in acute inferior myocardial infarction. Catheter. Cardiovasc. Interv..

[26] Mazimba S., Welch T.S., Mwansa H., Breathett K.K., Kennedy J.L., Mihalek A.D., Harding W.C., Mysore M.M., Zhuo D.X., Bilchick K.C. (2019). Haemodynamically Derived Pulmonary Artery Pulsatility Index Predicts Mortality in Pulmonary Arterial Hypertension. Heart Lung Circ..

[27] Kuwayama T., Morimoto R., Oishi H., Kato H., Kimura Y., Kazama S., Shibata N., Arao Y., Yamaguchi S., Hiraiwa H. (2020). Efficacy of Pulmonary Artery Pulsatility Index as a Measure of Right Ventricular Dysfunction in Stable Phase of Dilated Cardiomyopathy. Circ. J..

[28] Cesini S., Bhagra S., Pettit S.J. (2020). Low Pulmonary Artery Pulsatility Index Is Associated With Adverse Outcomes in Ambulatory Patients With Advanced Heart Failure. J. Card. Fail..

[29] Kochav S.M., Flores R.J., Truby L.K., Topkara V.K. (2018). Prognostic Impact of Pulmonary Artery Pulsatility Index (PAPi) in Patients With Advanced Heart Failure: Insights From the ESCAPE Trial. J. Card. Fail..

